# Supplementary material for: Rapid adaptive phenotypic change following colonization of a newly restored habitat
Source: Nat Commun. 2017 Jan 20;8:14159. doi: 10.1038/ncomms14159 (PMC5263874; doi:10.1038/ncomms14159)
Supplement: Supplementary Information — Supplementary Figures, Supplementary Table and Supplementary References [file ncomms14159-s1.pdf]

**Supplementary Table 1 | Microsatellite primer sequences**

| Locus | Primer sequence (5' → 3') | Reference |
|---|---|---|
| Aar4 | F: GATGACTAAGGTCTCTGGTGTG<br>R: GTTTGTGCATCAATTAGTCATG | Hansson *et al*. 2000[1] |
| Aar5 | F: GAGCTCTGTATGTGCGTG<br>R: TCTGAGTGGACTCAGGAGT | Hansson *et al*. 2000[1] |
| Aar8 | F: TAGTGATGCCCTGCTAGGTA<br>R: AAGTGCTCCTTAATATTTGGCA | Hansson *et al*. 2000[1] |
| Ase34 | F: GTTAATTCTTTTGGCCCTCAGC<br>R: GGAGACACCACACCAATGC | Richardson *et al*. 2000[2] |
| Ase58 | F: ATTCCAGGGATTGGGCAG<br>R: CTCAAAGCGAAATTGAGCAGT | Richardson *et al*. 2000[2] |
| Pca3 | F: GGTGTTTGTGAGCCGGGG<br>R: TGTTACAACCAAAGCGGTCATTTG | Dawson *et al*. 2000[3] |
| Pdoμ1 | F: TCTGGGCTGTTGCTATCAGAAGGA<br>R: GCAGGGCTGTCCTTTCAACACT | Neumann & Wetton 1996[4] |
| POCC2 | F: AACCACACTGAGTAAGCTGCTG<br>R: TTTAGCTCACCTTGCAAATGG | Bensch *et al*. 1997[5] |

**Supplementary Figure 1 | Variation in body mass and mean population fitness**

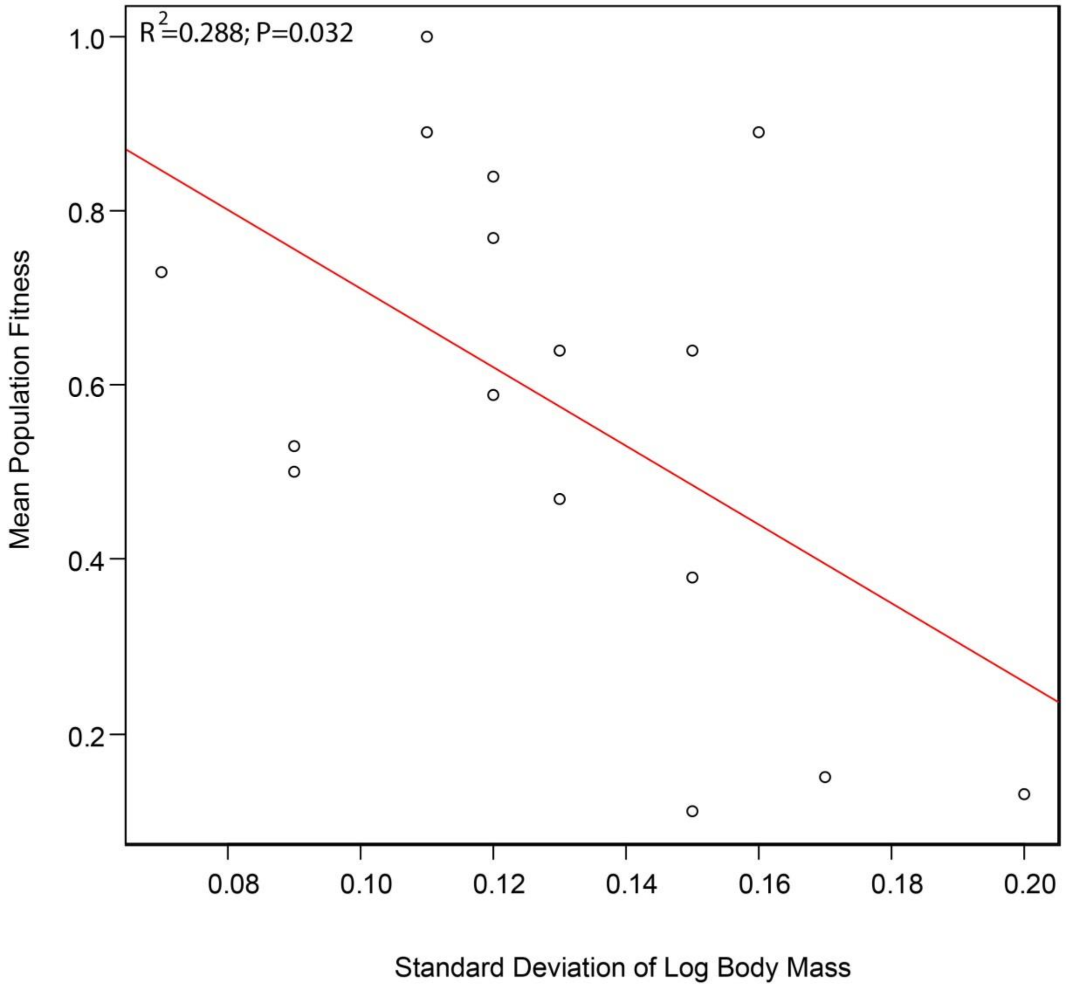


Linear regression of mean population fitness (proportion of breeding adults each year (*N* years = 16) against mean annual variation in body mass (as measured by the annual standard deviation). Mean annual variation in body mass decreases significantly with mean population fitness (Linear regression: $P = 0.032$), which suggest that body mass is under strong selection in this population. Overall, 28.8 % of the variation in mean population fitness can be explained by the mean annual variation in body mass.

**Supplementary Figure 2| Heritability of body mass**

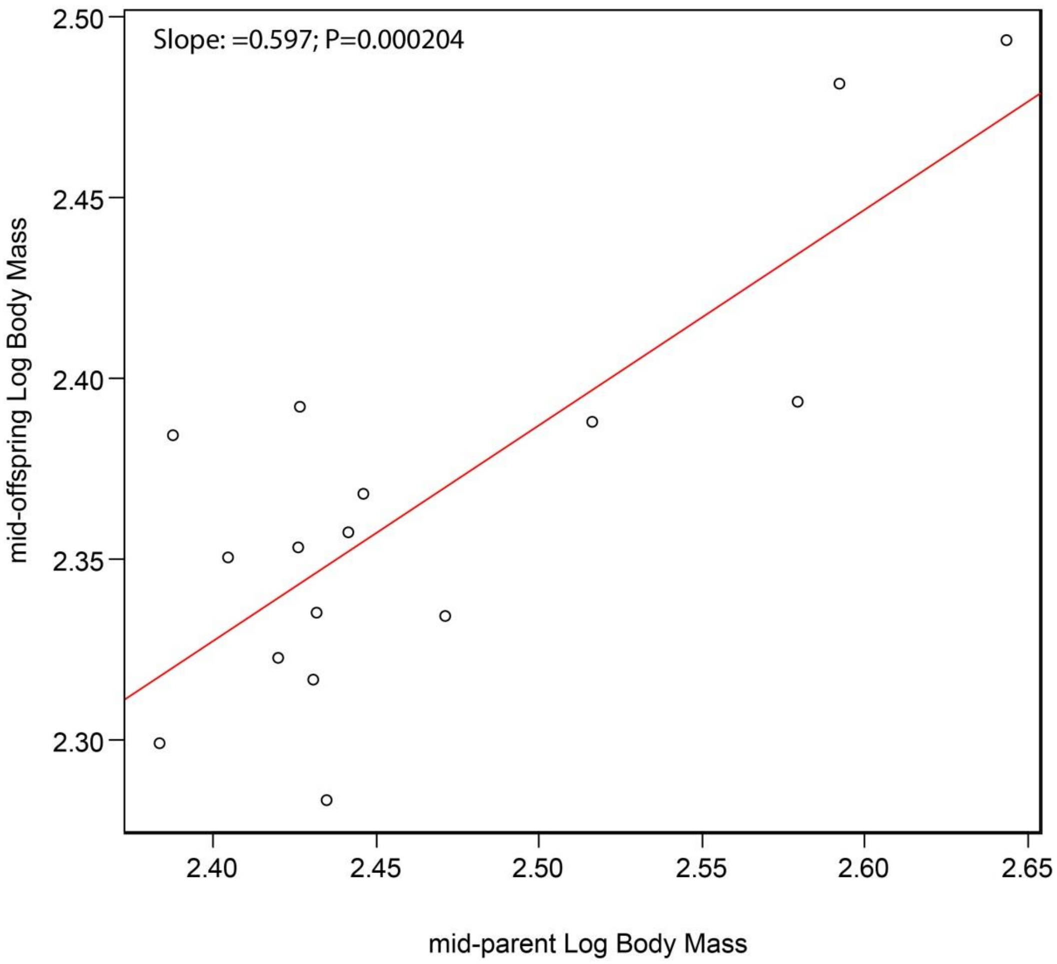


Linear regression of annual mid-parent log body mass against mid-offspring log body mass (*N* years = 16). Mid-parent and offspring body mass is significantly positively correlated with each other (Linear regression: $P = 0.0002$), which corresponds to a narrow-sense heritability of 0.597. This correlation remains significant when year is incorporated as a covariate in the linear model, to control for inter-annual variation in body mass which may reflect environmental variation (slope: 0.54; $P = 0.004$). This is consistent with the assumption that body mass has a significant heritable component in this population.

**Supplementary Figure 3 | Body mass of migrants from the source population**

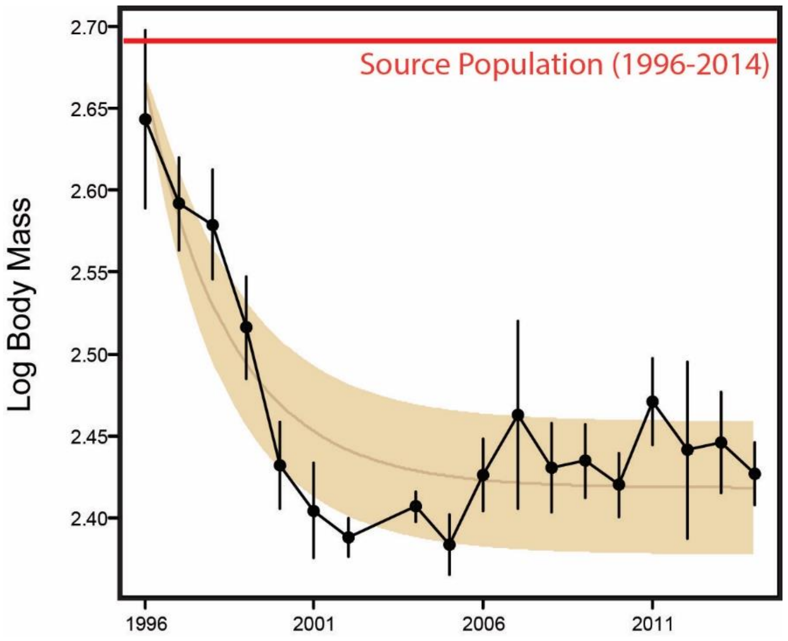


Between 1996 and 2014, 27 reed warblers were captured and measured outside the breeding season during migration (September-October). Presumably, they originate from Italy[6] and are only stopping over in Malta. These individuals are likely part of the original source population which colonized the restored habitat. Their average log body mass is equal to 2.69 (SE = 0.024; 95% CI: 2.66, 2.73), which is very close to the initial body mass of the Maltese population in 1996 ($N$ = 13, log body mass = 2.64) and is significantly different from the current local body mass ($N$ = 24, log body mass = 2.43), (one sample t-test: t = 11.19; $P$ < 0.001). This suggests that evolution of body mass has occurred *in situ* in Malta and is not the result of biased immigration.

**Supplementary References**

1. Hansson, B., *et al*. Increase of genetic variation over time in a recently founded population of great reed warblers (*Acrocephalus arundinaceus*) revealed by microsatellites and DNA fingerprinting. *Mol. Ecol.* **9,** 1529-1538 (2000).
2. Richardson, D. S., *et al*. Fifty Seychelles warbler (*Acrocephalus sechellensis*) microsatellite loci polymorphic in Sylviidae species and their cross-species amplification in other passerine birds. *Mol. Ecol.* **9**, 2225-2230 (2000).
3. Dawson, D. A., Hanotte, O., Greig, C., Stewart, I. R. K. & Burke, T. Polymorphic microsatellites in the blue tit *Parus caeruleus* and their cross-species utility in 20 songbird families. *Mol. Ecol.* **9**, 1941-1944 (2000).
4. Neumann, K. & Wetton, J. H. Highly polymorphic microsatellites in the house sparrow *Passer domesticus*. *Mol. Ecol.* **5**, 307-309 (1996).
5. Bensch, S. Price, T. & Kohn, J. Isolation and characterization of microsatellite loci in a *Phylloscopus* warbler. *Mol. Ecol*. **6**, 91-92 (1997).
6. Sultana, J. *et al.* The Breeding Birds of Malta. Birdlife Malta, Malta (2011).
